# Unexpected Rift Valley Fever Outbreak, Northern Mauritania

**DOI:** 10.3201/eid1710.110397

**Published:** 2011-10

**Authors:** Ahmed B. Ould El Mamy, Mohamed Ould Baba, Yahya Barry, Katia Isselmou, Mamadou L. Dia, Ba Hampate, Mamadou Y. Diallo, Mohamed Ould Brahim El Kory, Mariam Diop, Modou Moustapha Lo, Yaya Thiongane, Mohammed Bengoumi, Lilian Puech, Ludovic Plee, Filip Claes, Stephane de La Rocque, Baba Doumbia

**Affiliations:** Author affiliations: Centre National d’Etude et de Recherches Vétérinaires, Nouakchott, Mauritania (A.B O. El Mamy, Y. Barry, K. Isselmou, M.L. Dia);; Ministère du Développement Rural, Nouakchott (M.O. Baba, B. Doumbia);; Institut National de Recherches en Santé Publique, Nouakchott (B. Hampate, M.Y. Diallo, M.O. Brahim El Kory);; Institut Sénégalais de Recherches Agricoles, Dakar-Hann, Senegal (M. Diop, M.M. Lo, Y. Thiongane);; Food and Agriculture Organization of the United Nations Belvédère, Tunisia (M. Bengoumi, L. Puech);; Food and Agriculture Organization of the United Nations, Rome, Italy (L. Plee, F. Claes, S. de La Rocque);; Institute of Tropical Medicine, Antwerp, Belgium (F. Claes);; Centre International de Recherche Agronomique pour le Développement, Montpellier, France (S. de la Rocque)

**Keywords:** Rift Valley fever, outbreak, Mauritania, rainfall, oasis, camel, virus, dispatch

## Abstract

During September–October 2010, an unprecedented outbreak of Rift Valley fever was reported in the northern Sahelian region of Mauritania after exceptionally heavy rainfall. Camels probably played a central role in the local amplification of the virus. We describe the main clinical signs (hemorrhagic fever, icterus, and nervous symptoms) observed during the outbreak.

From late September through the beginning of October 2010, unprecedented rainfall created large ponds in the oases of the Saharan region of Adrar, northern Mauritania (Figure 1). Such rains had not been observed for decades; the local residents refer to 1956 (locally known as the “year of the fever”) to describe similar events. This climatic event translated into unusual growth of vegetation, attracting shepherds and pastoralists from remote areas, including the southern and southeastern regions of the country. It also favored high densities of mosquitoes, mainly from the genus *Culex* and *Anopheles* (*Cx. quinquefasciatus, An. pharoensis, An. protoriensis, Cx. poicilipes, An. gambiae, Aedes vexans, Cx. antenatus, An. rufipes, Mansonia uniformis, An. ziemani*); some of these species were known to be competent vector species for major arboviruses.

**Figure 1:**
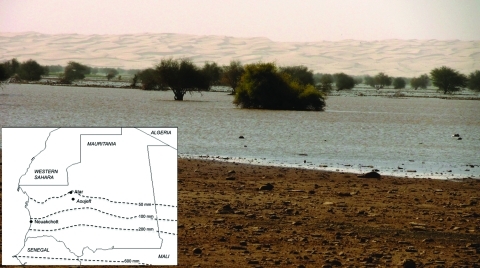
Lefrass Oasis, 30 km north of Atar, one of the main outbreak foci of an outbreak of Rift Valley fever in camels, northern Mauritania. Inset shows the location of Atar and Aoujeft and the isohyets (average during 1965−2002; source: Food and Agricultural Organization of the United Nations, Land and Water development Division).

A few weeks after these rains, severe outbreaks of malaria and Rift Valley fever (RVF) were reported in several oases (*graret*) of the Adrar region. Notably, the first probable reportable case in livestock was in a sick dromedary camel and occurred during the last week of October 2010 in the Aoujeft area; the camel’s signs were similar to those of pasteurellosis. The herdsman slaughtered the animal before it died but delayed the cutting up of the meat because of the remote location. Subsequently, the uncooked meat was shared within the extended family, and within a few days, several people died with intestinal and hemorrhagic symptoms. Health authorities requested testing for several pathogens, including Crimean-Congo hemorrhagic fever and RVF; results were positive for RVF. Although these persons likely did not become infected through the consumption of meat (the fall in pH during meat maturation rapidly destroys the virus) (*1*), the virus was obviously circulating intensively in this area at that time.

Two weeks after the index case, additional cases in camels, abortion storms in small ruminants, and human deaths (hemorrhagic fever, icterus, and nervous symptoms) were reported on a massive scale. At the end of December 2010, a total of 63 cases among humans, including 13 deaths, had been officially reported, but the true number is probably much higher due to the remoteness of the affected area. Of 14 initial blood samples from camels, 7 had positive test results by real-time reverse transcription PCR; the virus was isolated from 4 of those positive samples in the Laboratoire National d’Elevage et de Recherches Vétérinaires, Dakar, Senegal. The first serologic results obtained by the central veterinary laboratory using a competitive ELISA (ID Screen Rift Valley Fever Competition Multispecies ELISA, ID-Vet, Montpellier, France) indicated an immunoglobulin (Ig) M/IgG prevalence of 33% in camels and 44% in small ruminants, respectively. IgM titers (*2*) were as high as 45% in Adrar and even reached 54% in the eastern Inchiri area 2 weeks after the index case in the camel was observed (Table).

**Table:** Serologic data obtained from different regions, overall and per host species during Rift Valley Fever outbreak, northern Mauritania, September–October 2010*

Region	All samples, no. (%)				Small ruminant samples, no. (%)				Camel samples, no. (%)		
All samples	IgM/IgG positive	IgM positive†	All samples	IgM/IgG positive	IgM positive†	All samples	IgM/IgG positive	IgM positive†
Adrar	179	83 (46)	81 (45)		168	79 (47)	77 (46)		11	4 (36)	4 (36)
Brakna	17‡	3 (18)	0 (0)		2	0	0		10	3 (30)	0
Gorgol	8	2 (25)	2 (25)		8	2 (25)	2 (25)		0	0	0
Inchiri	57	32 (56)	31 (54)		57	32 (56)	31 (54)		0	0	0
Nouakchott	239	65 (27)	1 (0)		27	1 (*4*)	1 (4)		212	64 (30)	0
Nouadibou	46	20 (43)	8 (17)		0	0	0		46	20 (43)	8 (17)
Total	546	205 (37)	123 (23)		262	114 (43)	111		279	91 (33)	12 (*4*)

*Ig, immunoglobulin. †IgM-positive samples within the IgM/IgG-positive population. ‡Includes 5 cattle samples; all had negative test results.

Serologic evidence of RVF in camels is frequently reported (*3*), yet the description of clinical signs is rare (*4*). Some authors mention subclinical or mild forms (*5*) or even the capacity to carry the virus without clinical signs (*1*). In contrast, in the past, widespread abortion waves in camels were observed during RVF outbreaks in Kenya and Egypt and were associated with positive serologic test results (*6**,**7*). Furthermore, camels are suspected of playing a major role in the spread of RVF from northern Sudan to southern Egypt in 1977 (*8*). It should be noted that RVF virus was previously isolated from blood samples from healthy, naturally infected camels in Egypt and Sudan (*9**,**10*) and that experimental infections with RVF virus have induced no clinical signs in nonpregnant dromedaries (*3*).

During this outbreak, 2 clinical forms were observed in camels: a hyperacute form, with sudden death in <24 hours; and an acute form with fever, ataxia, ballooning, edema at the base of the neck, audible expiratory wheeze and ventral positional dyspnea, blood-tinged nasal discharge, icterus, severe conjunctivitis with ocular discharge and blindness, hemorrhages of gums and tongue, foot lesions, nervous symptoms, and abortions (Figure 2). When hemorrhagic signs developed, death usually occurred within a few days.

**Figure 2:**
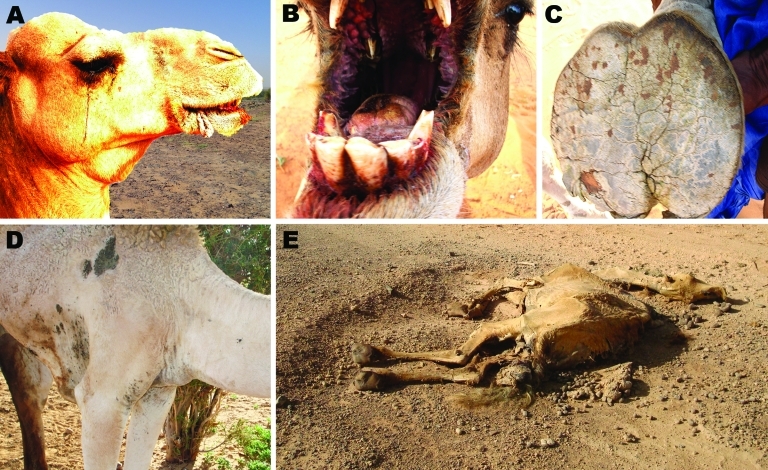
Observed clinical symptoms of Rift Valley fever in camels during field investigation in the Adrar region, northern Mauritania. A) Conjunctivitis and ocular discharge, hemorrhages of the gums, and edema of the trough; B) hemorrhages of gums and tongue; C) foot lesions (cracks in the sole) with secondary myasis; D) edema at the base of the neck; E) dead camel with sign of abortion, convulsions, and arching of the neck.

The current understanding of the outbreak is that the exceptional rainfall during September–October 2010 created highly favorable conditions for colonization and subsequent multiplication of competent vectors in these *grarets*. The virus was probably introduced rapidly through viremic animals transported by truck for grazing opportunities from various areas, including the south and southeastern regions of Mauritania where RVF is endemic (*11**,**12*). To cope with this outbreak, veterinary and public health authorities took appropriate control measures, including restriction of livestock movement, re-allocation of locust control teams for mass insecticide spraying, and risk communication and public awareness campaigns aimed at the population at risk.

## Conclusions

We report the unusual outbreak of RVF at a northern latitude and in an extremely arid region (although RVF has been reported in Egypt, where the Nile River helps spread the disease from the south). The high mortality rates and severe clinical signs observed among dromedary camels indicate that these animals played a major role in the epidemiology of this outbreak. The capacity of RVF-infected *Aedes* spp. eggs to survive in such an environment also needs further assessment. Indeed, increasing capacities for long-distance transportation, associated with increasing frequencies of extreme and hard-to-predict weather events, may create a challenging scenario for exotic diseases in general, and RVF in particular, to spread.

During the course of the outbreak in Adrar, the price of livestock decreased by 40%, which created an attractive opportunity for traders who potentially could further disseminate the virus. Also, the possible role of oases as relay points has for some time been seen as a major risk for the introduction of RVF in the Maghreb, where eco-climatic and entomologic conditions are favorable for its emergence. These possible risk factors and the unusual appearance of RVF in an arid region call for further strengthening of surveillance and sanitary capacities and policies.

## References

[1] Swanepoel R, Coetzer JAW. Rift Valley fever. In: Coetzer J, Tustin R, editors. Infectious diseases of livestock, 2nd ed. Oxford (UK): Oxford University Press; 2004. p. 1037–70.

[2] Paweska JT, Burt FJ, Anthony F, Smith SJ, Grobbelaar AA, Croft JE, IgG-sandwich and IgM-capture enzyme-linked immunosorbent assay for detection of antibody to Rift Valley fever in domestic ruminants. J Virol Methods. 2003;113:103–12. 10.1016/S0166-0934(03)00228-3 14553896

[3] Davies FG, Koros J, Mbugua H. Rift Valley fever in Kenya: the presence of antibodies to the virus in camels (*Camelus dromedarius*). J Hyg (Lond). 1985;94:241–4. 10.1017/S0022172400061441 3989285 PMC2129413

[4] Bird BH, Ksiazek TG, Nichol ST, MacLachlan NJ. Rift Valley fever virus. J Am Vet Med Assoc. 2009;234:883–93. 10.2460/javma.234.7.883 19335238

[5] Peters CJ, Meegan JM. Rift Valley fever in CRC handbook series in zoonoses. In: Beran G, editor. Boca Raton (FL): CRC Press; 1981. p. 403.

[6] Meegan JM, Hoogstraal H, Mousa MI. An epizootic of Rift Valley fever in Egypt in 1977. Vet Rec. 1979;105:124–5. 10.1136/vr.105.6.124 505918

[7] Scott GR, Roach RW, Cowdy NR, Coakley W. Rift Valley fever in camels. J Pathol Bacteriol. 1963;86:229–31. 10.1002/path.1700860131 13992529

[8] Eisa M, Obeid HMA, El Sawi ASA. Rift Valley fever in the Sudan. I—Results of field investigations of the first epizootic in Kosti District, 1973. Bull Anim Health Prod Afr. 1977;24:343–7.

[9] Eisa M. Rift Valley fever. OIE Technical Report Series. World Health Organization (Geneva). 1981;1:2–13.

[10] Imam ZEI, El-Karamany R, Darwish MA. An epidemic of Rift Valley fever in Egypt. 2. Isolation of the virus from animals. Bull World Health Organ. 1979;57:441–4. 314355 PMC2395804

[11] Thiongane Y, Martin V. Bulletin FAO de surveillance de la fièvre de la vallée du Rift en Afrique de l’Ouest (Mali, Mauritanie, Sénégal), no. 1. Rome: Food and Agricultural Organization of the United Nations; 2000.

[12] Zeller HG, Fontenille D, Traore-Lamizana M, Thiongane Y, Digoutte JP. Enzootic activity of Rift Valley fever virus in Senegal. Am J Trop Med Hyg. 1997;56:265–72. 9129528 10.4269/ajtmh.1997.56.265

